# Importance of human peritoneal mesothelial cells in the progression, fibrosis, and control of gastric cancer: inhibition of growth and fibrosis by tranilast

**DOI:** 10.1007/s10120-017-0726-5

**Published:** 2017-05-24

**Authors:** Hiroto Saito, Sachio Fushida, Shinichi Harada, Tomoharu Miyashita, Katsunobu Oyama, Takahisa Yamaguchi, Tomoya Tsukada, Jun Kinoshita, Hidehiro Tajima, Itasu Ninomiya, Tetsuo Ohta

**Affiliations:** 1 Department of Gastroenterological Surgery, Division of Cancer Medicine, Graduate School of Medical Science, Kanazawa University, 13-1 Takara-machi, Kanazawa, Ishikawa 920-8641 Japan; 2 Center for Biomedical Research and Education, School of Medicine, Kanazawa University, Kanazawa, Ishikawa 920-8641 Japan

**Keywords:** Tranilast, Gastric cancer, Fibrosis, Human peritoneal mesothelial cells, TGF-β

## Abstract

**Background:**

Scirrhous gastric cancer is an intractable disease with a high incidence of peritoneal dissemination and obstructive symptoms (e.g., ileus, jaundice, and hydronephrosis) arising from accompanying marked fibrosis. Microenvironmental interactions between cancer cells and cancer-associated fibroblasts are the suggested cause of the disease. We elucidated the mechanisms of tumor growth and fibrosis using human peritoneal mesothelial cells (HPMCs) and investigated the effects of tranilast treatment on cells and a xenograft mouse model of fibrosis.

**Methods:**

HPMCs were isolated from surgically excised omentum and their interaction with MKN-45 gastric cancer cells was investigated using co-culture. Furthermore, a fibrosis tumor model was developed based on subcutaneous transplantation of co-cultured cells into the dorsal side of nude mice to form large fibrotic tumors. Mice were subsequently treated with or without tranilast.

**Results:**

The morphology of HPMCs treated with transforming growth factor (TGF)-β1 changed from cobblestone to spindle-type. Moreover, E-cadherin was weakly expressed whereas high levels of α-smooth muscle actin expression were observed. TGF-β-mediated epithelial–mesenchymal transition-like changes in HPMCs were inhibited in a dose-dependent manner following tranilast treatment through inhibition of Smad2 phosphorylation. In the mouse model, tumor size decreased significantly and fibrosis was inhibited in the tranilast treatment group compared with that in the control group.

**Conclusions:**

Tranilast acts on the TGF-β/Smad pathway to inhibit interactions between cancer cells and cancer-associated fibroblasts, thereby inhibiting tumor growth and fibrosis. This study supports the hypothesis that tranilast represents a novel strategy to prevent fibrous tumor establishment represented by peritoneal dissemination.

## Introduction

Despite decreased incidence in recent decades, an estimated 951,600 new gastric cancer cases and 723,100 deaths were reported globally in 2012 [1]. Peritoneal dissemination, common among scirrhous gastric cancer patients, is a critical factor in its poor prognosis [2], and is characterized by diffusely infiltrating and proliferating cancer cells accompanied by extensive stromal fibrosis in the peritoneal cavity. Peritoneal dissemination induces obstructive symptoms, including ileus, jaundice, and hydronephrosis, as a result of fibrosis. Multimodal treatment, including systemic chemotherapy [3], intraperitoneal chemotherapy [4, 5], and aggressive surgery [6], has not improved the clinical outcomes for gastric cancer patients with peritoneal dissemination. Therefore, new treatment strategies are needed to target tumor proliferation and fibrosis in peritoneal dissemination of gastric cancer.

The epithelial–mesenchymal transition (EMT) of epithelial cells, an important mechanism in tissue fibrosis, is characterized by the loss of epithelial cell characteristics such as cell–cell adhesion [7]. Furthermore, mesenchymal markers such as α-smooth muscle actin (α-SMA) are expressed by newly formed fibroblasts called myofibroblasts, and are considered specific markers for EMT [8]. Transforming growth factor (TGF)-β1 has been shown to induce tissue fibrosis via EMT, mediated by the Smad pathway in various organs, including the peritoneum [9]. It is a common initiator of EMT, and several studies have demonstrated that TGF-β1 may be a key mediator of fibrosis [10]. TGF-β1 secreted by gastric cancer cells [11] and cancer-associated fibroblasts (CAFs) [12] strongly promotes disease progression and may be responsible for the poor prognosis associated with gastric cancer [13, 14]. TGF-β activates type II TGF-β receptors (TβR-II), which phosphorylate type I TGF-β receptors (TβR-I) [15]. The activated TGF-β receptors in turn stimulate the phosphorylation of Smad2/3, which subsequently associates with Smad4 and translocates to the nucleus to form transcriptional complexes [16, 17]. TGF-β1 is therefore a key factor in the development of myofibroblasts from fibroblasts and a variety of precursor cells.

Some studies have reported that tumor stroma, which often contains stromal fibroblasts, plays a role in cancer progression and metastasis [18]. CAFs comprise a major part of the cellular components of the tumor microenvironment and are derived from resident stromal fibroblasts and fibroblast-like cells such as bone marrow-derived cells [19, 20]. It has been proposed that epithelial cells can transform into CAFs [21], which acquire myofibroblast properties including induced expression of α-SMA and decreased expression of E-cadherin. Moreover, CAFs play an important role in the malignant progression of several cancers such as lung [22], pancreatic [23], and esophageal cancer [24]. Previous reports suggest that fibroblasts play an important role in the progression and growth of scirrhous gastric cancers [25, 26]. CAFs may support the malignant progression of tumors by promoting tumor growth, survival, invasion, and metastasis through the secretion of stroma-modulating growth factors such as TGF-β1, hepatocyte growth factor (HGF) [27], vascular endothelial growth factor (VEGF) [28], and stromal cell derived factor-1 (SDF-1) [20, 29]. Moreover, fibroblast activation protein (FAP), a transmembrane serine protease highly expressed in CAFs and present in >90% of human epithelial neoplasms, supports tumorigenesis mechanisms such as tissue remodeling and immunosuppression [30].

We have previously shown that human peritoneal mesothelial cells (HPMCs), a monolayer of mesothelial cells that lines the peritoneal cavity and internal organs, are activated by TGF-β1 and can undergo EMT to adopt the fibroblast or myofibroblast-like phenotype referred to as CAFs [31]. We have also demonstrated the interaction between gastric cancer cells and HPMCs using a fibrous xenograft model that imitates the cancer microenvironment of peritoneal dissemination [31]. Taken together, these findings suggest that antifibrotic agents may improve the prognosis of patients with scirrhous gastric cancer.

We have previously reported that low-dose paclitaxel (PTX) [32], protein-bound polysaccharide K (PSK) [33], and angiotensin II receptor blockers (ARB) [11] have the potential for use as antifibrotic agents in the treatment of gastric cancer patients with peritoneal dissemination. Tranilast (*N*-[3,4-dimethoxycinnamonyl]-anthranilic acid), an orally administered drug of low toxicity, has been used clinically in Japan as an antiallergic and antifibrotic agent as it inhibits fibroblast growth and the production of chemical mediators from fibroblasts in keloid tissue [34]. In some experimental animal studies, tranilast has been shown to prevent the c of coronary arteries after percutaneous transluminal coronary angioplasty (PTCA) [35], suppress neointima development after stenting [36], inhibit fibrosis in postmyocardial infarction and collagen production in cardiac fibroblasts by attenuating myocardial TGF-β1 expression [37], and attenuate TGF-β-induced collagen synthesis by cardiac fibroblasts in diabetic cardiomyopathy [38]. Other animal studies have indicated that tranilast prevents renal tubulointerstitial fibrosis by inhibiting EMT of normal rat kidney proximal tubular epithelial cells and may ameliorate tubulointerstitial fibrosis, especially in chronic cyclosporine nephrotoxicity, by inhibiting the TGF-β/Smad pathway [39]. These findings have led us to hypothesize that tranilast may effectively suppress tissue fibrosis during peritoneal dissemination of gastric cancer.

We have also previously established that a mouse xenograft model using the gastric cancer cell line MKN-45 in co-culture with HPMCs promotes tumor proliferation and fibrosis [31]. In the established mouse xenograft models, we demonstrated that HPMCs have a latent ability to function as CAFs and contribute to tumor fibrosis in the tumor microenvironment.

In this study, we investigated whether tranilast inhibits the effects of TGF-β-induced EMT in HPMCs by inhibiting the TGF-β/Smad pathway, and whether fibrosis can be attenuated in our established fibrotic tumor model using the gastric cancer cell line MKN-45 in co-culture with HPMCs.

## Materials and methods

### Cell lines and cell culture

HPMCs were isolated from surgical specimens of the human omentum, as previously described [39, 40]. Written informed consent for use of these specimens, as required by the Institutional Review Board at Kanazawa University, Japan, was obtained from patients undergoing elective abdominal surgery. Small segments of omentum were surgically resected under sterile conditions and were incubated in pre-warmed phosphate-buffered solution (PBS) containing 0.125% trypsin/EDTA (Gibco/Invitrogen, USA) for 30 min at 37 °C. The resulting suspension was passed through a 100-μm pore nylon mesh (Becton–Dickinson, Japan) to remove undigested tissue fragments and centrifuged at 1500 rpm for 5 min. The collected cells were cultured in RPMI-1640 medium (Gibco/Invitrogen) supplemented with 30% heat-inactivated fetal bovine serum (FBS; Nichirei Bioscience Inc., Japan), 100 IU/mL penicillin, 100 mg/mL streptomycin (Gibco/Invitrogen), and 2 mM glutamine (Nissui Pharmaceutical Co. Ltd., Japan). The cells were seeded into gelatin-coated 75-cm^2^ flasks (BD BioCoat, USA) and cultured in 10 mL of medium at 37 °C under a humidified atmosphere of 5% CO_2_. Subconfluent HPMCs were trypsinized with 0.125% trypsin/EDTA before use and employed at passages 1–3 in all experiments.

For the subsequent experiments, cells were used during the second or third passage after primary culture. HPMCs that were potentially contaminated with endothelial cells or fibroblasts at the time of harvest were not used. Donors had not received chemotherapy or radiation treatment prior to surgery, and had no evidence of peritoneal inflammation and/or malignancy. Homogeneous HPMCs from a different donor were used for each experiment.

The gastric cancer cell lines MKN-7, MKN-45, and MKN-74 were purchased from the Japanese Collection of Research Bioresources (JCRB) Cell Bank (Osaka, Japan). MKN-7 cells were established from metastatic foci to lymph nodes with a microscopic phenotype of a well-differentiated tubular adenocarcinoma (tub1) [41, 42]. MKN-45 cells were derived from a hepatic metastatic tumor with a microscopic phenotype of a solid type of poorly differentiated adenocarcinoma (por1) [41, 42]. MKN-74 cells were established from a hepatic metastatic tumor with a microscopic phenotype of a moderately differentiated tubular adenocarcinoma (tub2) [41, 42]. Cells were maintained in RPMI-1640 medium supplemented with 10% FBS. OCUM-2MD3, a cell line derived from a human scirrhous gastric cancer and with high peritoneal-seeding activity, was kindly provided by the Department of Surgical Oncology of Osaka City University of Medicine [43, 44]. OCUM-2MD3 cells were seeded into 75-cm^2^ dishes (Becton Dickinson, Tokyo, Japan) and cultured in 10 mL Dulbecco’s modified Eagle’s medium (DMEM; Life Technologies, Tokyo, Japan) supplemented with 10% heat-inactivated FBS, 100 IU/mL penicillin, 100 mg/mL streptomycin, 2 mM glutamine, and 0.5 mM sodium pyruvate at 37 °C in a humidified atmosphere of 5% CO_2_. We routinely evaluated cell cultures for mycoplasma infection, and negative results were obtained with a PCR mycoplasma test kit (Promokine, Heidelberg, Germany).

### Chemicals

Tranilast (*N*-[3,4-dimethoxycinnamonyl]-anthranilic acid) was obtained from Tokyo Chemical Ind. Co. (Japan) and diluted to the required concentrations in RPMI-1640 and DMEM medium for the in vitro study and dissolved in 1% NaHCO_3_ solution for the in vivo study. TGF-β1 was purchased from Sigma–Aldrich, Inc. (USA) and reconstituted in RPMI-1640 medium at appropriate concentrations.

### Mouse xenograft model

All animal experiments were performed according to Kanazawa University’s standard guidelines. Female immunocompromised BALB/c-nu/nu mice (Charles River Laboratories Inc., Japan) at 4–6 weeks of age were maintained in a sterile environment. MKN-45 cells were co-cultured with an equivalent number of HPMCs for 5 days, and a total of 5 × 10^6^ cells in 100 μL of RPMI-1640 were subcutaneously implanted into the dorsal side of each mouse on day 0. Four groups of six mice each were established: MKN-45 cells alone (5 × 10^6^ cells), with or without tranilast; and MKN-45 cells co-cultured with HPMCs, with or without tranilast. Beginning on day 7, mice were administered 200 mg/kg/day of tranilast daily by gavage. Animals were carefully monitored and tumors were measured every 4 days. At day 28, the mice were sacrificed and the tumors were removed for immunohistochemical examination. The tumor volume (*V*) was calculated according to the formula *V* = *AB* ^2^/2, where *A* is the length of the major axis and *B* is the length of the minor axis.

### Histological and immunohistochemical examination

Tumor specimens were fixed in 10% neutral buffered formalin and embedded in paraffin. Sections were stained with hematoxylin and eosin (H&E) and Azan stain for the assessment of fibrosis, and the expression of E-cadherin antibody (H-108, rabbit polyclonal IgG, diluted 1:100; Santa Cruz Biochemistry, Inc.) and α-smooth muscle actin (α-SMA; 1A4, mouse monoclonal IgG, diluted 1:100; DakoCytomation, Denmark) was assessed immunohistochemically. Deparaffinized sections were pretreated by autoclaving in 10% citric acid buffer (pH 8.0) at 120 °C for 15 min. Following treatment with protein blocking serum (DakoCytomation, Kyoto, Japan) for 10 min, sections were incubated with primary antibody at 4 °C overnight. EnVision polymer solution [horseradish peroxidase (HRP), DakoCytomation] was then applied for 1 h and signal was developed in 0.02% 3,3′-diaminobenzidinetetrahydrochloride (DAB) solution containing 0.1%. Sections were then lightly counterstained with hematoxylin and examined using a fluorescence microscope (Olympus, Tokyo, Japan). The degree of fibrosis was calculated as the percentage of fibrosis within the whole section in all samples using a BZ-9000 BZII microscope (Keyence, Osaka, Japan).

### Phase contrast microscopy

HPMCs were seeded into 100-mm tissue culture dishes at 5 × 10^4^ cells in RPMI-1640 growth medium with 10% FBS. HPMCs in culture were then treated with TGF-β1 (5 ng/mL) for 72 h, with and without pre-treatment with 200 or 400 μM tranilast for 1 h, and morphological changes were visualized by phase contrast microscopy. Images were captured using an inverted microscope (Nikon Corp., Japan).

### Immunofluorescence

For visualization of FAP, E-cadherin, and α-SMA in HPMCs, cells were grown on four-well collagen type I-coated culture slides (BD BioCoat) and fixed when preconfluent in a 1:1 mixture of methanol and acetone for 10 min. Briefly, slides were immersed in methanol containing 0.3% H_2_O_2_ for 30 min, blocked with 3.3% normal goat serum in PBS, and incubated with FAP antibody (ab53066, rabbit polyclonal IgG: diluted 1: 100; Abcam, Tokyo, Japan), E-cadherin antibody (H-108, rabbit polyclonal IgG; diluted 1:100; Santa Cruz Biotechnology, Inc., USA), and α-SMA (1A4, mouse monoclonal IgG; diluted 1:100; DakoCytomation, Denmark) at 4 °C overnight. Following three washes in PBS, immunoreactivity was visualized by incubating the slides with an anti-mouse IgG antibody conjugated with Alexa Fluor^®^ 488 and an anti-rabbit IgG antibody conjugated with Alexa Fluor^®^ 546 (1:400; Molecular Probes/Invitrogen, USA) for 1 h at room temperature. Cells were then incubated for 5 min with Hoechst 33258 for nuclear staining and mounted with propyl gallate containing phenylenediamine under glass coverslips. The slides were observed using an immunofluorescence microscope (BX50/BS-FLA; Olympus, Japan).

### MTT assay

The effect of tranilast on the proliferative capacities of MKN-7, MKN-45, MKN-74, OCUM-2MD3, and HPMCs was quantified using the 3-(4,5-dimethylthiazol-2-yl)-2,5-diphenyltetrazolium bromide (MTT) assay. MKN-7, MKN-45, MKN-74, and HPMCs were seeded in 96-well plates at 4 × 10^3^ cells per well in RPMI-1640 growth medium with 10% FBS, and OCUM-2MD3 cells were seeded in DMEM medium with 10% FBS. The cells were incubated overnight at 37 °C in a humidified environment containing 5% CO_2_. Following incubation, supernatant was discarded and replaced with fresh serum-free medium and various concentrations of tranilast (50, 100, 200, or 400 μM) were added. At 48 h post-treatment, supernatant was discarded, MTT solution was added to each well (final concentration, 500 μg/mL), and plates were incubated at 37 °C for 3 h. The supernatant was then removed and 150 μL of DMSO (Wako, Japan) were added. The absorbance of the solution in each well was measured at 535 nm using a microplate reader (model 550; Bio-Rad, Japan). Cell viability was calculated as viability = (absorbance of experimental wells)/(absorbance of control wells). All experiments were repeated at least three times.

### Western blotting

Approximately 5 × 10^6^ cells were lysed in RIPA buffer containing 1% protease inhibitor cocktail (Sigma–Aldrich, Inc.). Protein from each sample was loaded onto 12.5% SDS-PAGE gels and subjected to electrophoresis. Proteins were then transferred to PVDF membranes (Bio-Rad, USA) and blocked with blocking solution (0.1% Tween-20; EZ Block ATTO Corporation, Japan) at room temperature for 30 min. Blots were incubated overnight at 4 °C with each primary antibody (see below). The blots were then incubated for 1 h with appropriate HRP-conjugated secondary antibodies and visualized using an ECL Plus western blotting detection system (GE Healthcare Japan Ltd., Japan) and a light-capture system (ATTO). To ensure equal protein loading, β-actin levels were determined using an anti-β-actin monoclonal antibody (AC-15, mouse monoclonal IgG, diluted 1:10,000; Sigma–Aldrich, Inc.). The following primary antibodies were used: E-cadherin (H-108, rabbit polyclonal IgG, diluted 1:1000; Santa Cruz Biotechnology, Inc.), α-SMA (1A4, mouse monoclonal IgG, diluted 1:5000; Santa Cruz Biotechnology, Inc.), Smad2 (YZ-13, mouse monoclonal IgG, diluted 1:1000; Santa Cruz Biotechnology, Inc.), phospho-Smad2 (ser467, rabbit polyclonal IgG; Santa Cruz Biotechnology, Inc.).

### Statistical analysis

All data are expressed as the mean ± SD. Statistical analyses were conducted using SPSS statistical software, version 23 (SPSS Inc., Chicago, IL, USA). Comparisons of drug effects were made using one-way analysis of variance (ANOVA) or Student’s *t* test. A *p* value < 0.05 was considered to indicate a statistically significant difference.

## Results

### Effect of tranilast on morphological changes in HPMCs following treatment with TGF-β1

**Fig. 1:**
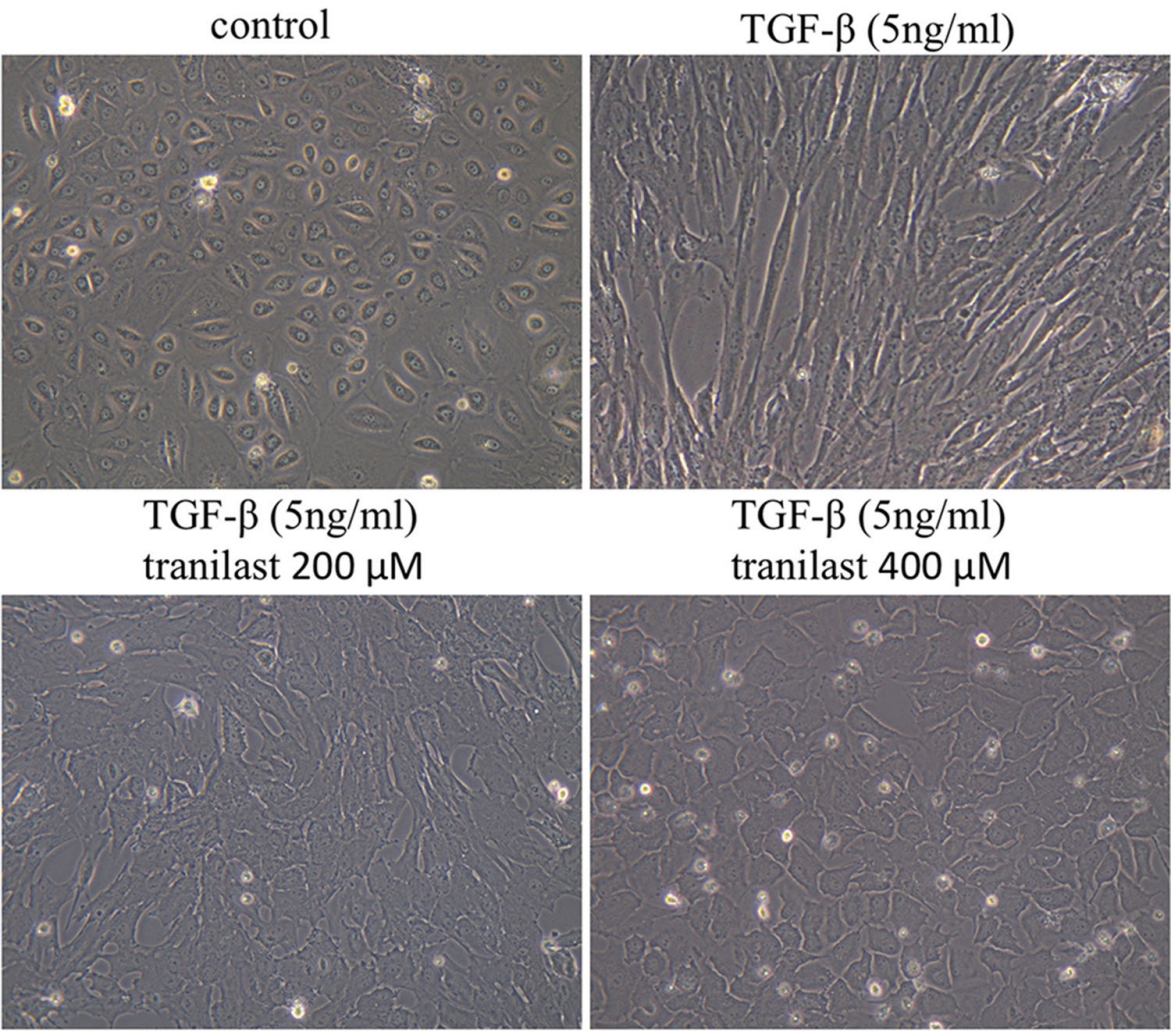
Phase contrast microscopy of morphological changes in HPMCs. HPMCs cultured in control medium exhibited a cobblestone-like growth pattern. HPMCs treated with TGF-β1 (5 ng/mL) for 72 h exhibited a spindle fibroblast-like morphology. Pretreatment with 200 and 400 μM tranilast prevented spindle-like changes induced by TGF-β1 and resulted in rounding of the cells. Original magnification × 400

Untreated HPMCs exhibited a polygonal and cobblestone-like growth pattern. In contrast, HPMCs treated with TGF-β1 (5 ng/mL) showed the spindle-shaped morphology characteristic of fibroblasts. Pretreatment with tranilast (200 or 400 μM) prevented the induction of these morphological changes by TGF-β1 (Fig. 1).

### FAP expression and EMT marker in HPMCs

**Fig. 2:**
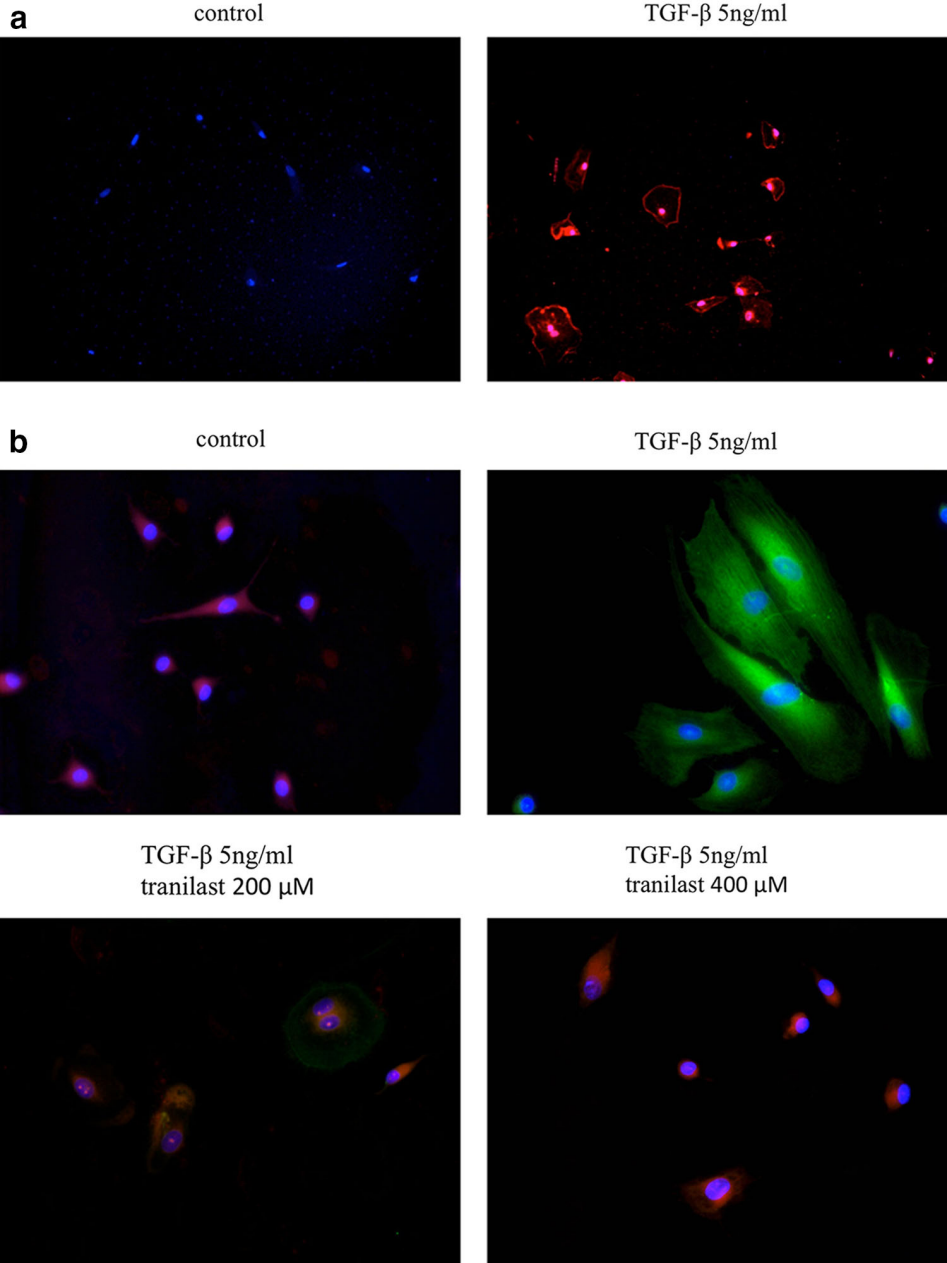
**a** Representative photomicrographs of immunofluorescence staining for FAP. FAP expression was observed on the membranes of HPMCs treated with TGF-β1. Original magnification × 200. **b** Representative photomicrographs of immunofluorescence staining for E-cadherin (*red*) and α-SMA (*green*). HPMCs treated with TGF-β1 showed increased expression of α-SMA and decreased expression of E-cadherin, whereas pretreatment with 200 or 400 μM tranilast suppressed α-SMA expression and reinstated the E-cadherin expression. Original magnification × 400

**Fig. 3a–c:**
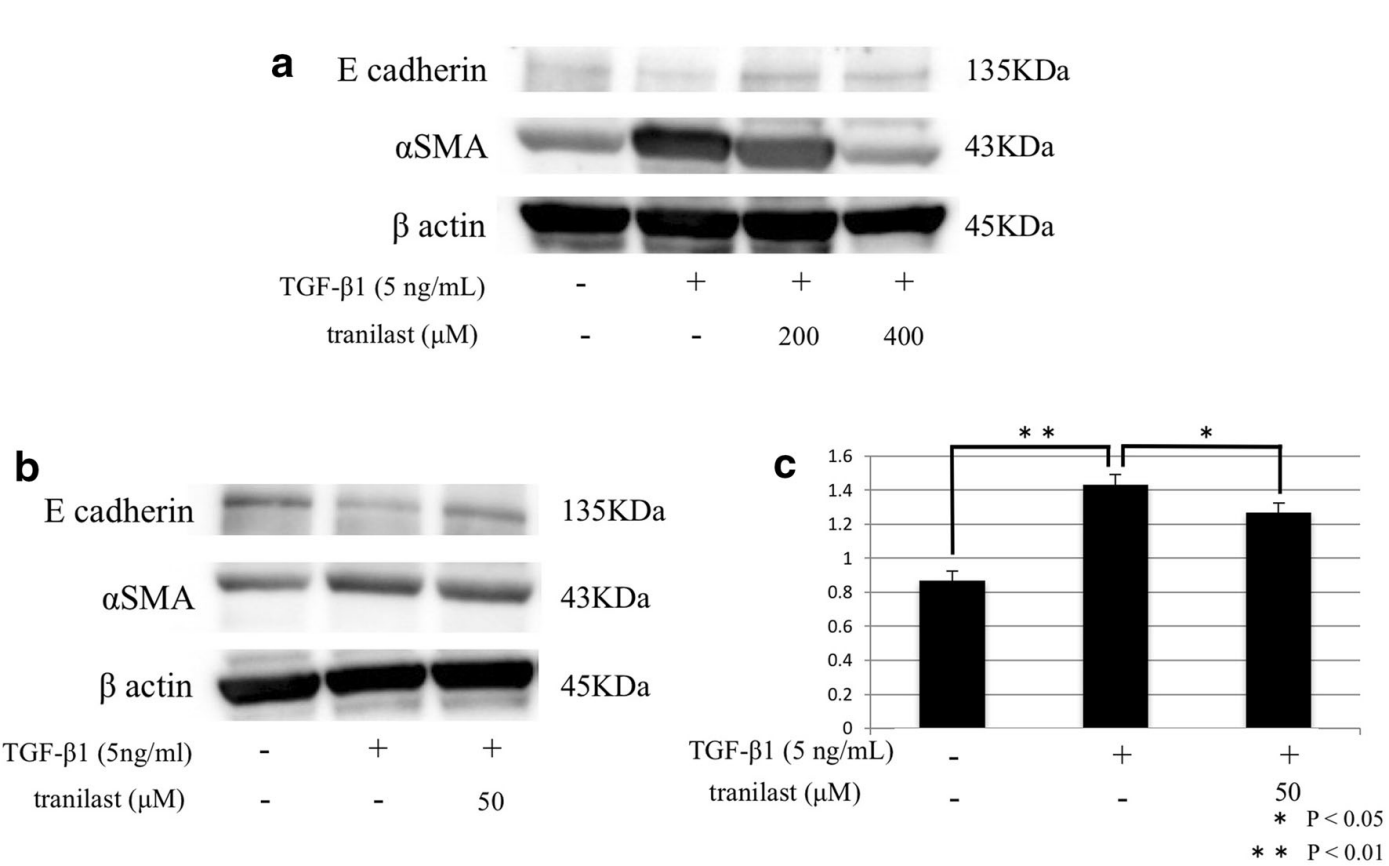
Western blot analysis of E-cadherin and α-SMA expression in HPMCs. **a** α-SMA expression was higher in HPMCs treated with TGF-β than in control cells, and this increase was inhibited by pretreatment with 200 or 400 μM tranilast. E-cadherin expression was attenuated in HPMCs treated with TGF-β, and the expression was reinstated by pretreatment with 200 or 400 μM tranilast. **b** Tranilast (50 μM) inhibited α-SMA expression and enhanced E-cadherin expression. **c** Densitometry analyses of α-SMA expression following treatment with 50 μM tranilast were performed in three independent experiments; data are expressed as the mean ± SD. *p < 0.05, **p < 0.01

FAP expression was observed by immunofluorescence staining on the membranes of HPMCs treated with TGF-β1 (Fig. 2a). Immunofluorescence staining of HPMCs treated with TGF-β1 showed that the mesenchymal marker α-SMA was upregulated in the cytoplasm and that E-cadherin expression decreased. Pretreatment of HPMCs with tranilast prior to TGF-β1 administration prevented the increase in α-SMA expression in the cytoplasm and reinstated the expression of E-cadherin in a dose-dependent manner (Fig. 2b). Western blotting confirmed the immunofluorescence findings of increased α-SMA protein expression and decreased E-cadherin expression in HPMCs treated with TGF-β1, and that these effects were inhibited by pretreatment with tranilast in a dose-dependent manner (Fig. 3a). Furthermore, treatment with 50 μM tranilast inhibited the EMT changes in HPMCs treated with TGF-β1 (Fig. 3b, c).

### Effect of tranilast TGF-β/Smad signaling in HPMCs

**Fig. 4:**
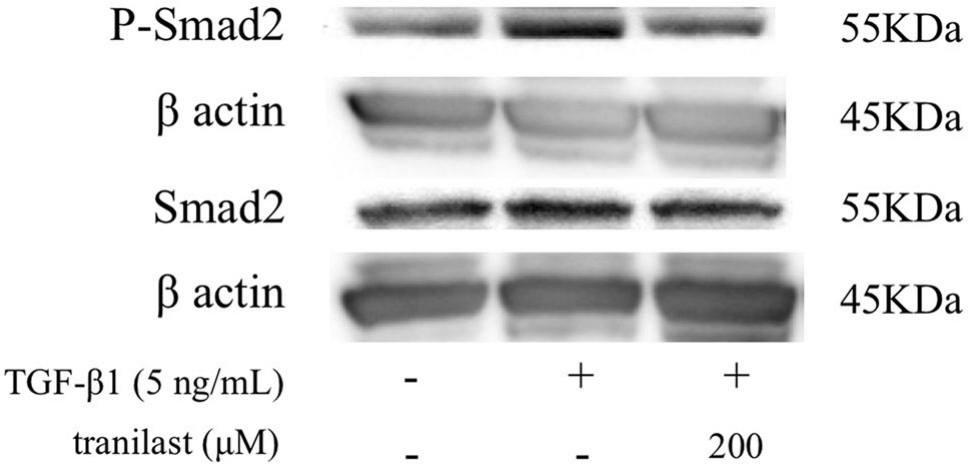
Western blot analysis for the effect of tranilast on Smad2 phosphorylation in HPMCs. Smad2 phosphorylation was observed in cells treated with TGF-β1 for 1 h, whereas pretreatment with 200 μM tranilast inhibited phosphorylation. Smad2 expression was not affected in control cells or in TGF-β1 and tranilast-treated groups

To investigate TGF-β/Smad pathway interactions, Smad2 phosphorylation was assessed in HPMCs. Western blotting showed that TGF-β1 induced the phosphorylation of Smad2 and pretreatment with 200 μM tranilast inhibited the TGF-β1-induced phosphorylation of Smad2, consistent with the blockade of TGF-β signaling (Fig. 4).

### Effect of tranilast on the proliferation of HPMCs and gastric cancer cell lines

**Fig. 5a–e:**
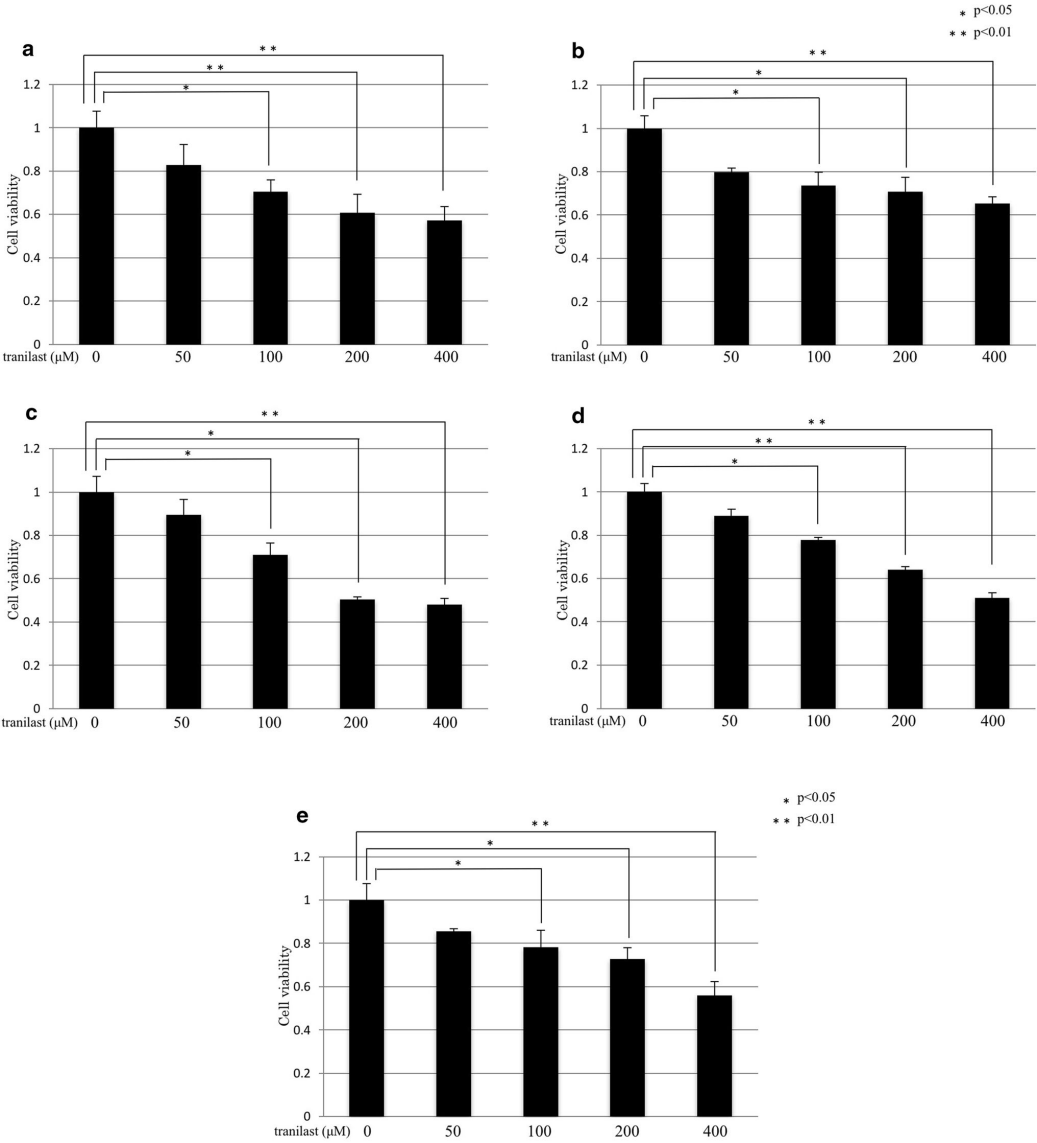
Inhibitory effects of tranilast on the proliferation of gastric cancer cell lines and HPMCs. MTT assays showed that 50 μM tranilast did not significantly affect the growth of gastric cancer cell lines and HPMCs. **a** MKN-7, **b** MKN-45, **c** MKN-74, **d** OCUM-2MD3, **e** HPMCs. Results are the mean ± SD of three experiments. *p < 0.05, **p < 0.01

Tranilast attenuated the proliferation of gastric cancer cell lines (MKN-7, MKN-45, MKN-74, and OCUM-2MD3) and HPMCs in a dose-dependent manner, with no significant effect on growth at a dose of 50 μM (Fig. 5a–e).

### Effect of tranilast in subcutaneous xenograft models

**Fig. 6a–e:**
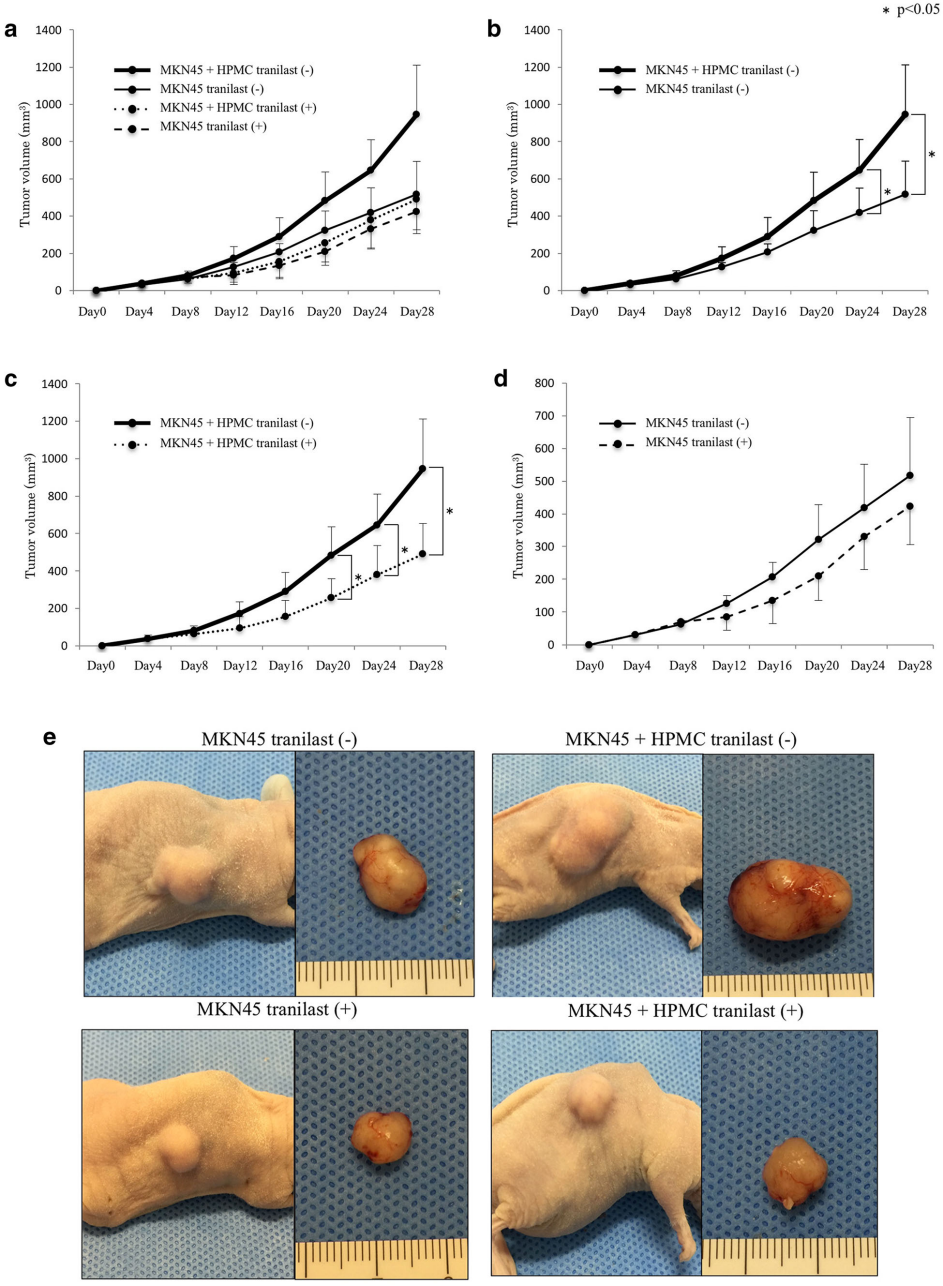
Subcutaneous xenograft model to investigate the antifibrotic effects of tranilast. Time course of tumor growth at day 28 in **a** all four groups, **b** MKN-45 group without tranilast versus MKN-45 and HPMCs co-culture group without tranilast, **c** MKN-45 and HPMCs co-culture group with or without tranilast, **d** MKN-45 group with or without tranilast. Results are expressed as the mean ± SD (*n* = 6). **e** Representative images show the macroscopic appearance of tumors at day 28. MKN-45 tranilast (−): MKN-45 without tranilast, MKN-45 tranilast (+): MKN-45 with 200 mg/kg tranilast, MKN-45 + HPMCs tranilast (−): co-culture of MKN-45 and HPMCs without tranilast, MKN-45 + HPMCs tranilast (+): co-culture of MKN-45 and HPMCs with 200 mg/kg tranilast. *p < 0.05

To determine whether tranilast had antiproliferative and antifibrotic properties in vivo, a dose of 200 mg/kg/day was administered orally to female nude mice with tumor xenografts. The time course of relative subcutaneous tumor volume is shown in Fig. 6a.

At 28 days post-transplantation, the mean relative volumes of tumors arising from MKN-45 cells co-cultured with HPMCs and without the administration of tranilast were significantly larger than those of tumors derived from MKN-45 cells alone (*p* = 0.022) (Fig. 6b). This result was consistent with that of our previous study [29]. In addition, tumors derived from MKN-45 cells co-cultured with HPMCs were significantly smaller in the tranilast treatment group compared with those in the untreated group (*p* = 0.011) (Fig. 6c). In mice transplanted with MKN-45 cells alone, tumor size decreased in the tranilast treatment group, but the change was not significant (Fig. 6d).

### Histological and immunohistochemical examination of xenograft tumors

**Fig. 7a–e:**
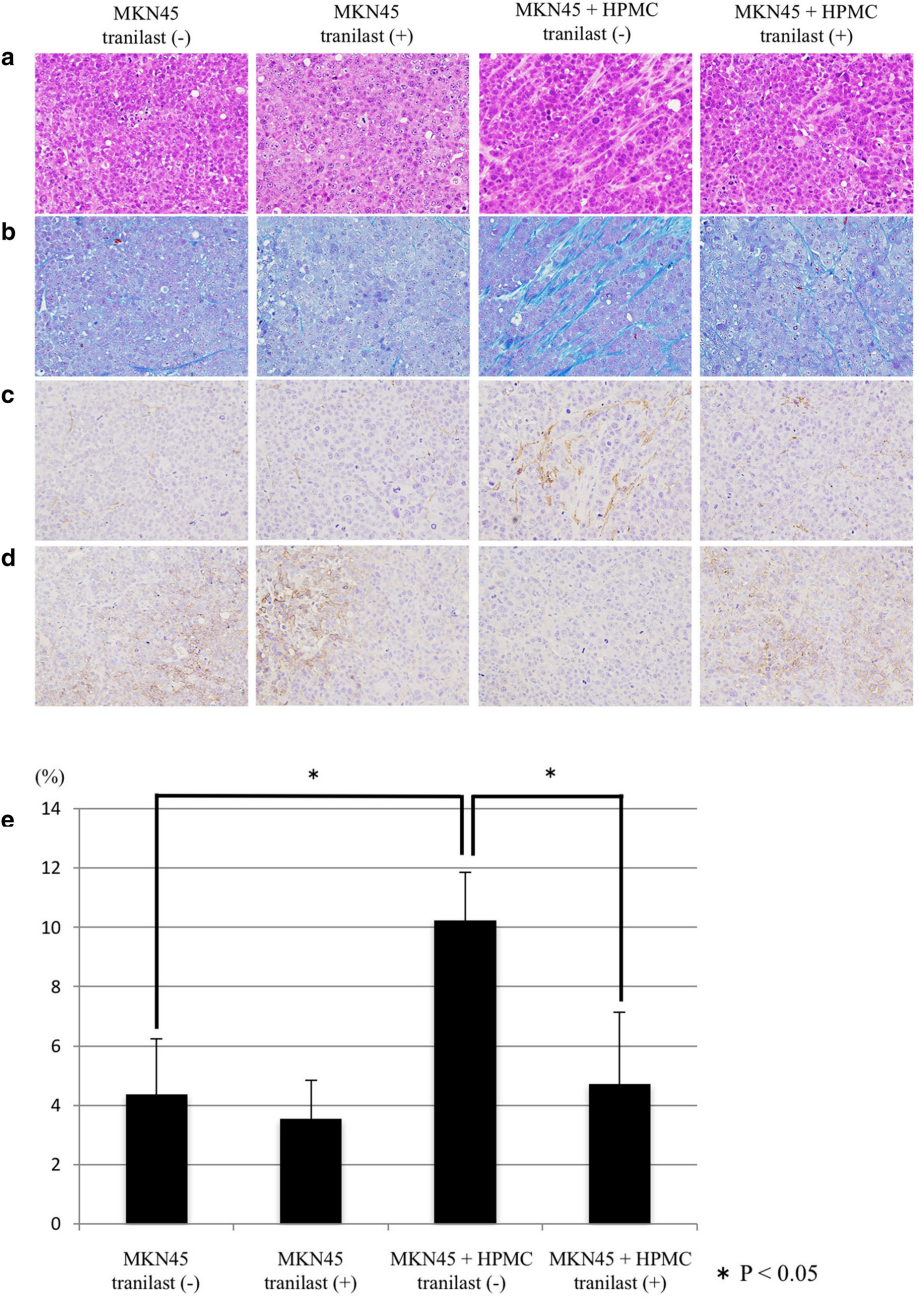
Microscopic views of mouse xenograft tumors. **a** Histological examination using H&E staining. **b** Fibrous tissue was determined by Azan staining. **c** Immunohistochemical examination of α-SMA and **d** E-cadherin (original magnification × 200). **e** The fibrotic area was measured and is shown as a percentage (fibrotic area/whole section area) of that in **b**. *p < 0.05

Tumors derived from implanted MKN-45 and HPMCs co-cultures had larger areas of fibrosis, enhanced α-SMA expression, and decreased E-cadherin expression (Fig. 7a–d). However, tumors from xenograft mice treated with tranilast showed decreased α-SMA expression and increased E-cadherin expression compared to that reported for untreated tumors. In the co-cultured tumor groups, Azan staining revealed a significantly lower degree of fibrosis in the tranilast-treated group compared to that observed for the untreated group (*p* = 0.026) (Fig. 7e).

## Discussion

In this study, we investigated the effects of low doses of tranilast (<100 μM) on cytotoxicity in HPMCs and gastric cancer cells. We have previously suggested that TGF-β1-mediated activation of HPMCs during EMT is one factor in the development of CAFs and can promote peritoneal fibrosis [31]. Our findings suggest that tranilast can significantly suppress fibrosis and tumor proliferation in a subcutaneous xenograft mouse model. Therefore, tranilast may have the potential to attenuate the activation of HPMCs induced by TGF-β1 in peritoneal dissemination.

We have previously reported that HPMCs transform into myofibroblast-like cells following exposure to TGF-β1, indicating that HPMCs are one source of CAFs [31]. FAP, a member of the serine protease family, is selectively expressed in stromal fibroblasts associated with epithelial cancers [30]. A key characteristic of CAFs is the expression of FAP, and we confirmed that FAP is expressed on the membranes of HPMCs treated with TGF-β. Direct and indirect interactions between CAFs and cancer cells are important for promoting their proliferation and invasion [45]. CAFs and cancer cells indirectly interact through paracrine signaling mediated by soluble factors, such as fibroblast growth factor, platelet-derived growth factor, vascular endothelial growth factor, and TGF-β1 [19, 46]. TGF-β1, which is secreted by both gastric cancer cells and CAFs, is regarded as one of the key molecules responsible for cell migration and invasion in the peritoneal dissemination of scirrhous gastric cancer [47, 48]. Our results show that HPMCs treated with TGF-β1 acquired a spindle-shaped morphology as opposed to a cobblestone-like growth pattern, and showed increased expression of α-SMA. Regulation of TGF-β signaling is important for inhibiting fibrosis and decreasing the invasiveness and metastasis of gastric cancer. Some studies have reported that a TGF-β-neutralizing antibody and a TGF-β receptor kinase inhibitor can inhibit EMT and attenuate stromal fibrosis [32, 33, 47]. We demonstrated that tranilast suppresses TGF-β signaling through the inhibition of Smad2 phosphorylation. We also demonstrated that tranilast inhibits TGF-β-induced EMT-like changes in HPMCs and may also inhibit fibrosis in our established tumor model. Inhibition of the TGF-β/Smad pathway by tranilast, as described previously, is important for the prevention of tumor fibrosis.

One factor associated with poor prognosis among scirrhous gastric cancer patients is peritoneal dissemination with marked fibrosis, which progresses in a multistep process [49]. First, cancer cells attach to HPMCs in the peritoneal cavity and release various factors, including TGF-β1 [31, 50], which can stimulate HPMCs and induce EMT-like morphological changes [9, 31]. Such changes promote the adhesion of cancer cells to the submesothelial basement membrane [9]. Moreover, activated HPMCs are a source of CAFs through transformation to a myofibroblast-like phenotype. These CAFs can acquire the ability to invade the submesothelial basement membrane, together with cancer cells, to facilitate highly fibrotic changes and proliferation [31]. This process is supported by the observation that peritoneal dissemination can develop in any organ containing HPMCs.

We evaluated tranilast as an antifibrotic agent in this study. Tanaka et al. previously reported that the* C*_max_ of tranilast at the therapeutic dose in humans (300 mg/day) is estimated to be 100 μM in peripheral blood [35]. Waseda et al. previously reported a tissue concentration of 30 μM for oral tranilast (300 mg/day) in clinical therapy [51]. In mice, one study reported a tissue concentration of tranilast of about 75 μM following the consecutive oral administration of 200 mg/kg/day [52]. In this study, we found that tranilast at doses greater than 50 μM and less than 100 μM had the effect of attenuating CAF function but did not exert an antiproliferative effect in vitro. We therefore used an oral dose of 200 mg/kg/day in our xenograft model. We found no statistical difference in mouse xenograft tumor size between the groups with tumors derived from MKN-45 cells alone, regardless of whether 200 mg/kg/day tranilast treatment was applied (Fig. 6d). This result indicates that 200 mg/kg/day tranilast may not have an antitumor effect in mice. However, in our fibrous xenograft model where tumors were derived from co-cultured MKN-45 cells and HPMCs, tumor size and fibrosis significantly decreased in the tranilast-treated group. The effect of low-dose tranilast may attenuate the function of CAFs derived from HPMCs and prevent tumor fibrosis and growth. Tranilast has been reported to exhibit antitumor properties in previous studies at doses above 100 μM [53, 54], although tranilast concentrations above 100 μM are not clinically relevant. Our findings show that doses of tranilast that approximate clinical dosing regimens may have inhibitory effects on the function of CAFs. Ohshio et al. also demonstrated that low-dose tranilast potentially inhibits CAF function by suppressing the induction of immune suppressor cells such as regulatory T cells and myeloid-derived suppressor cells [55]. Tranilast was shown to be effective at doses of 50 μM in vitro and 200 mg/kg/day in vivo—doses that are only approximately 1.7- to 2.5-fold higher than clinical doses. Therefore, clinical doses of tranilast have the potential to prevent tumor fibrosis.

A limitation of this study is the possibility that tranilast delivery differed between the subcutaneous fibrotic tumor model and the orthotopic implantation model. Further studies are therefore required to investigate whether low-dose tranilast inhibits tumor growth and fibrosis in the xenograft model of peritoneal dissemination with organ invasion and fibrosis, similar to clinical observations.

In conclusion, we have shown that tranilast can suppress the TGF-β/Smad pathway by inhibiting Smad2 phosphorylation in HPMCs treated with TGF-β1, and can significantly decrease growth and stromal fibrosis in a mouse fibrous xenograft model. Tranilast has been widely used clinically as an antiallergic drug and for the treatment of keloid disorder without any serious side effects. This study supports the hypothesis that tranilast represents a novel strategy to prevent fibrous tumor establishment represented by peritoneal dissemination. We believe that this is the first report of the antifibrotic effects of low-dose tranilast in a fibrosis tumor model.
